# Status of Systemic Oxidative Stress during Therapeutic Hypothermia in Patients with Post-Cardiac Arrest Syndrome

**DOI:** 10.1155/2013/562429

**Published:** 2013-08-26

**Authors:** Kenji Dohi, Kazuyuki Miyamoto, Kenichiro Fukuda, Shunsuke Nakamura, Munetaka Hayashi, Hirokazu Ohtaki, Seiji Shioda, Tohru Aruga

**Affiliations:** ^1^Department of Emergency and Critical Care Medicine, Showa University Fujigaoka Hospital, 1-30 Fujigaoka, Aoba-ku, Yokohama, Kanagawa 227-8501, Japan; ^2^Department of Anatomy, Showa University, Tokyo, Japan; ^3^Department of Emergency and Critical Care Medicine, Showa University, Tokyo, Japan

## Abstract

Therapeutic hypothermia (TH) is thought to be due to the downregulation of free radical production, although the details of this process remain unclear. Here, we investigate changes in oxidative stress and endogenous biological antioxidant potential during TH in patients with post-cardiac arrest syndrome (PCAS). Nineteen PCAS patients were enrolled in the study. Brain temperature was decreased to the target temperature of 33°C, and it was maintained for 24 h. Patients were rewarmed slowly (0.1°C/h, <1°C/day). The generation of reactive oxygen metabolites (ROMs) was evaluated in plasma samples by d-ROM test. Plasma antioxidant capacity was measured by the biological antioxidant potential (BAP) test. Levels of d-ROMs and BAP levels during the hypothermic stage (33°C) were suppressed significantly compared with pre-TH induction levels (*P* < 0.05), while both d-ROM and BAP levels increased with rewarming (33–36°C) and were correlated with brain temperature. Clinical monitoring of oxidative stress and antioxidant potential is useful for evaluating the redox state of patients undergoing TH after PCAS. Additional therapy to support the antioxidant potential in the rewarming stage following TH may reduce some of the observed side effects associated with the use of TH.

## 1. Introduction

Preresuscitation therapies, including basic life support and advanced cardiac life support, have contributed to the increased survival rates after cardiac arrest [1]. On the other hand, the global ischemia-reperfusion injury frequently observed after cardiac arrest with the return of spontaneous circulation (ROSC) causes serious damage to the brain and other organs. To this extent, in a recent study of 24,132 patients in the United Kingdom who were admitted to critical care units after cardiac arrest, the in-hospital mortality rate was 71% [2]. In 2008, the International Liaison Committee on Resuscitation (ILCOR) defined whole body ischemia/reperfusion injury following ROSC as the condition of Post-Cardiac Arrest Syndrome (PCAS) [3–5]. The pathophysiological characteristics of PCAS were defined as follows: (1) post-cardiac arrest brain injury, (2) post-cardiac arrest myocardial dysfunction, (3) systemic ischemia/reperfusion (I/R) response, and (4) persistent precipitating pathology [3, 4]. It has been reported that the systemic I/R response results in the production of reactive oxygen species (ROS) and oxidative stress [6–8], with oxidative stress being one of the main factors responsible for secondary damage and inflammation after I/R [9, 10].

Therapeutic hypothermia (TH) is well known as a cornerstone of early postresuscitation care in cardiac arrest survivors [3, 11, 12]. One of the targets of TH is the control of oxidative stress in I/R-induced injury [13, 14]. TH is associated not only with benefits, but also with serious side effects, including immunosuppression with increased infection risk, cold diuresis and hypovolemia, electrolyte disorders, insulin resistance, impaired drug clearance, and mild coagulopathy [15]. 

In previous reports, we studied the effects of free radical oxidative stress under various neuropathological conditions [16–18] and revealed that TH suppressed alkoxyl radical production after traumatic brain injury (TBI) [16]. The purpose of the present study was to clarify the effect of TH on oxidative stress and on the antioxidant potential of patients with PCAS.

## 2. Material and Methods

### 2.1. Patients and Intensive Care

The study was performed in accordance with the Declaration of Helsinki, and the study protocol was approved by the ethics committee of Showa University School of Medicine (Protocol no. 217).

Nineteen patients who had been subjected to cardiopulmonary resuscitation (CPR) and subsequently admitted to the Showa University Hospital Emergency Center (Tokyo, Japan) were enrolled in this study. Demographic and clinical parameters of the patients are presented in Table 1.

### 2.2. Treatments

Cooling blankets, rapid cold fluid infusion (up to 1000 mL saline, human plasma products, or dextrose-free plasma expanders), and/or cold gastric lavage were the cooling techniques used during the induction phase. The aim was to achieve a target temperature of 33°C within 6 h of the onset of CPA (cardio pulmonary arrest). The desired temperature was to be maintained for 24 h, mainly using surface cooling blankets. The patient was rewarmed at a rate of 0.1°C/h at not more than 1°C/day, with core body temperature maintained at <37.5°C for 7 days after the onset of cardiac arrest. Core body temperature was measured by a thermistor coupled to an intracranial pressure (ICP) monitoring probe (CAMINO, model 110-4BT). Internal jugular venous and bladder temperatures were also measured. In addition to critical neurological care, an arterial catheter and ICP monitoring probe were inserted to maintain the hemodynamic status and ICP at the following levels: mean arterial pressure (MAP) >80 mmHg, cardiac index (CI) >2.5 L/min/m^2^, systemic vascular resistance index (SVRI) 800–1200 dynes/sec/cm^−5^/m^2^, ICP <20 mmHg, and cerebral perfusion pressure (CPP) >60 mmHg. The partial pressures of arterial oxygen (PaO_2_) and carbon dioxide (PaCO_2_) were maintained at >100 mmHg and 30–40 mmHg, respectively. Anticonvulsants were administered. The sedation protocol used specified midazolam (0.2–0.4 mg/kg/h) and neuroleptic analgesia (1 *μ*g/kg/h fentanyl). Vecuronium (0.05 mg/kg/h) was also used in each sedation protocol during the induction and maintenance phases as deemed necessary. Vasopressors (dopamine, dobutamine) were continuously infused if necessary. Sedatives and analgesics were usually tapered off once the patients had been rewarmed to 36°C. Muscle relaxant use was stopped when shivering had disappeared, which usually occurred during the maintenance phase. Muscle relaxants were restarted as deemed necessary. Anticoagulant and antiplatelet therapies were started after confirmation of hemostasis after catheters and ICP probe insertion. 

### 2.3. Blood Sampling

Whole blood sampling was performed by catheters placed in the internal jugular bulb pre-TH (1st day), during the cooling stage (2nd day), and during the rewarming stage at each 1°C point.

All blood analyses were performed using a free radical analyzer system (FRAS4, Diacron, Grosset, Italy) that included a spectrophotometric device reader and a thermostatically regulated minicentrifuge; kits for the measurement of different blood parameters were optimized to the FRAS4 system according to the manufacturer's instructions. All analyses were performed immediately after blood collection to avoid falsely high or low results. To analyze the plasma ROM levels, diacron-reactive oxygen metabolites (d-ROMs) tests were used (Diacron, Grosset, Italy), while the measurement of antioxidant capacity and thiol-antioxidant capacity was performed with biological antioxidant potential (BAP) tests (Diacron, Grosset, Italy).

### 2.4. Hydroperoxide Assay (d-ROM Test)

The d-ROM test reflects the level of organic hydroperoxides present in the plasma, which in turn reflects the level of free radicals from which they were formed. When the samples are dissolved in an acidic buffer, the hydroperoxides react with transition metal ions liberated from the proteins in the acidic medium and are converted to alkoxy and peroxy radicals. These newly formed radicals oxidize an additive aromatic amine (*N,N*-diethyl-*para*-phenylenediamine) and cause the formation of a relatively stable colored cation radical that is spectrophotometrically detectable at 505 nm. The results are expressed in arbitrary units (U CARR; derived from the name of the chemist (Carratelli) who invented the test) [19–23]. The linearity range of the d-ROMs test is between 50 and 500 U CARR; for values up to 500 U CARR, a sample dilution is required. The intra-assay coefficient of variation for this assay is 2.1%, while the interassay coefficient of variation is 3.1%. It has been experimentally established that 1 U CARR corresponds to 0.08 mg of H_2_O_2_/dL. Reference values for healthy subjects are between 250 and 300 U CARR (20.08–24.00 mg H_2_O_2_/dL) [19].

### 2.5. Biological Antioxidant Potential Assay (BAP Test)

The BAP test provides an estimate of the global antioxidant capacity of blood plasma, measured as its reducing potential against ferric ions. When the sample is added to a colored solution (obtained by mixing a ferric chloride solution with a thiocyanate derivative solution), decoloration results. The intensity of the decoloration is spectrophotometrically detectable at 505 nm and is proportional to the ability of plasma to reduce ferric ions [20–24]. Photometric reading was employed to assess the intensity of decoloration. A lyophilized human control serum with known antioxidant activity (*μ*M) was used to periodically calibrate the FRAS4 system. Reference values for healthy subjects are above 2200 *μ*M [25].

### 2.6. Statistical Analysis

Data are presented as the mean ± SD. d-ROM and BAP test results at each time point were compared using repeated ANOVA tests. Correlations were evaluated using the Pearson product-moment correlation coefficient. A comparison between the correlations was made using Fisher's method. Values of *P* < 0.05 were taken to represent statistically significant differences for these analyses.

## 3. Results

**Figure 1:**
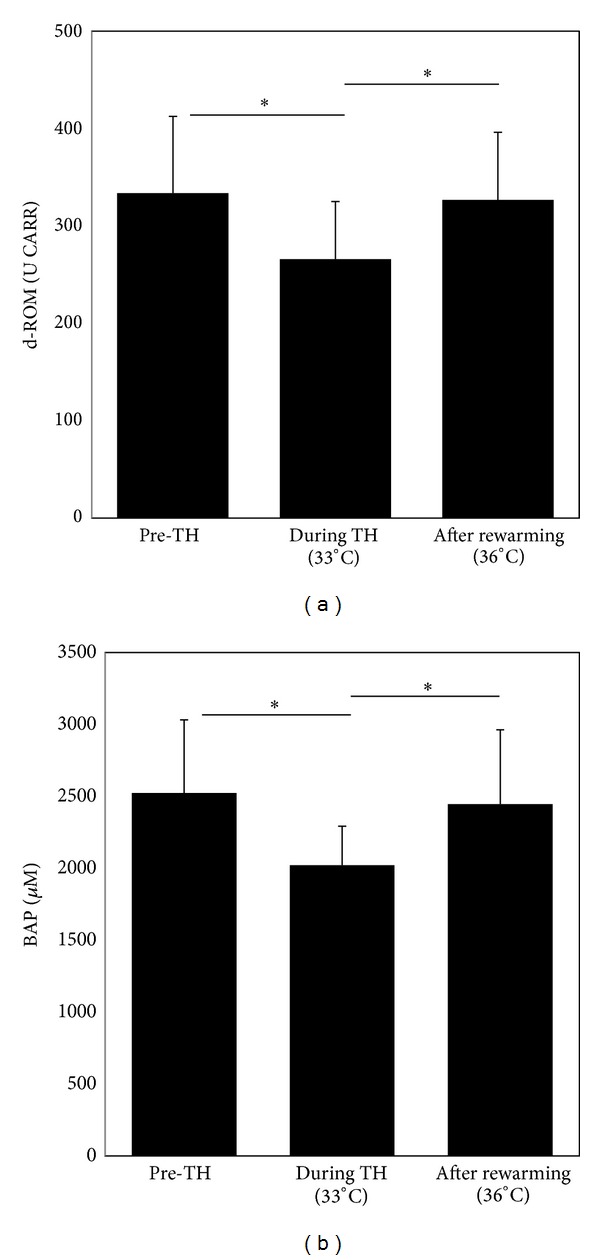
Plasma levels of derivatives of reactive oxygen metabolites (d-ROM) (a) and biological antioxidant potential (BAP) (b) in patients with PCAS prior to the induction of therapeutic hypothermia (pre-TH), during hypothermia (33°C), and after rewarming (36°C). Values are expressed as the mean, with the error bar representing the standard deviation. *Significantly different between TH and other groups, *P* ≤ 0.05.

**Figure 2:**
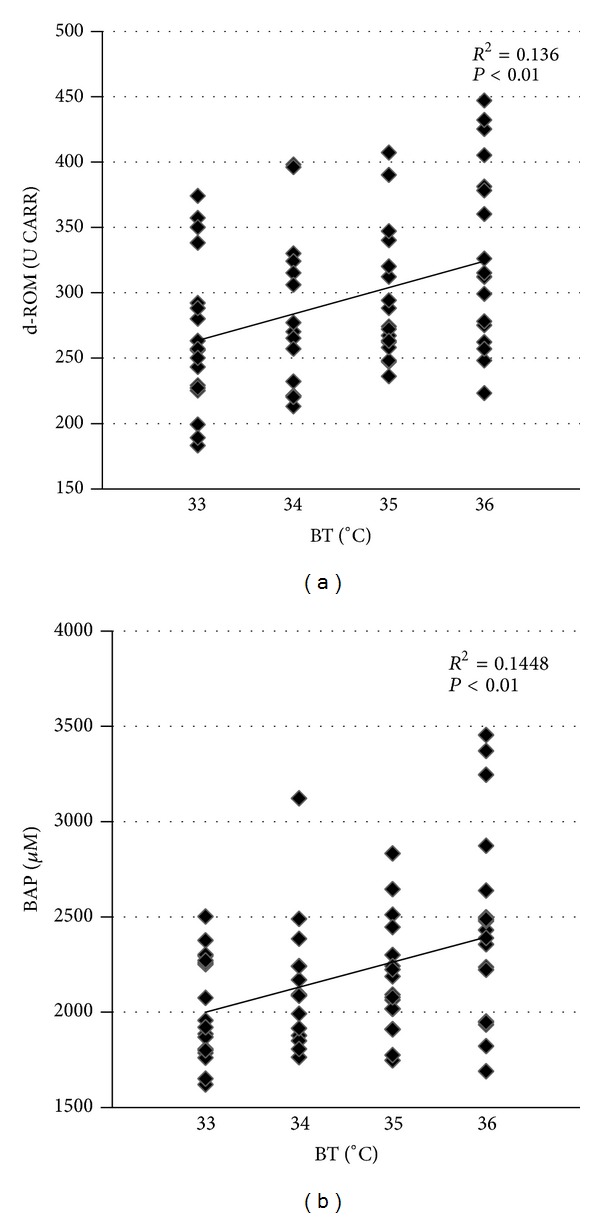
The Pearson product-moment correlation analysis between brain temperature and derivatives of reactive oxygen metabolites (d-ROM) (a) and biological antioxidant potential (BAP) (b) during the rewarming phase. The Pearson product-moment correlation coefficient (*R* ^2^) is indicated for each relationship.

Levels of d-ROMs in pre-TH patients (333.6 ± 79.0 U CARR) were higher than reference values from healthy subjects (250−300 U CARR). After the induction of TH, d-ROM levels during the hypothermic phase (33°C) were significantly suppressed compared with the pre-TH levels (266.7 ± 57.6 U. Carr versus 333.6 ± 79.0 U CARR, *P* < 0.05; Figure 1(a)). During the rewarming phase, d-ROM levels again increased in line with rewarming from 33 to 36°C (Figure 1(b)). BAP levels were also significantly suppressed during the hypothermic phase (2021.4 ± 272.2 *μ*M versus 2523.0 ± 550.5 *μ*M, *P* < 0.05; Figure 2(a)). As for the d-ROM results, BAP levels also increased with the rewarming from 33 to 36°C (Figure 2(b)). In this way, d-ROM and BAP levels were significantly correlated with brain temperature during the rewarming phase (*P* < 0.05).

### 3.1. Case Result

**Figure 3:**
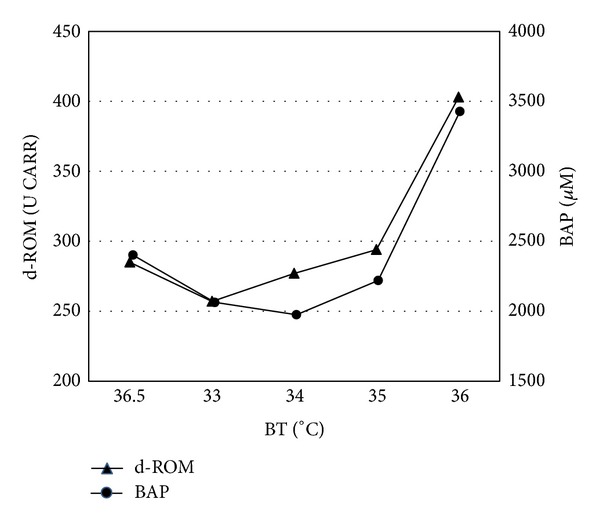
A Case of a 66-year-old male patient with PCAS. Derivatives of reactive oxygen metabolites (d-ROMs) and biological antioxidant potential (BAP) with therapeutic hypothermia are shown. Kinetics of d-ROM and BAP are similar except for levels at 34°C.

The case of a patient with PCAS is presented in Figure 3. A 66-year-old male with a known medical history of hypertension, atrial fibrillation, and hyperlipidemia complained of the sudden onset of chest pain while he was at home. On arrival the rescue team found an unconscious patient with CPA. After several minutes of cardiopulmonary resuscitation and a single defibrillation with automated external defibrillators (AEDs), spontaneous circulation was restored. His level of recovery of consciousness was incomplete upon admission to our hospital (E1 V1 M5/Glasgow Coma Scale). TH (33°C, 24 h) was performed using a cooling blanket with the patient placed under sedation. The target brain temperature (33°C) was accomplished within 4 h of the cardiac arrest. Brain temperature was maintained at 33°C for 24 h, following which the patient was rewarmed at a rate of <1°C/day until a core body temperature just inferior to 37.5°C had been obtained and maintained thereafter for 7 days. Severe stenosis and obstruction of the coronary arteries were not observed by cardioangiography, but a diagnosis of vasospastic angina was made. d-ROM and BAP levels were monitored throughout the TH treatment, with the results shown in Figure 3. Both the oxidative stress and the antioxidant potential decreased in response to the induction of TH. During rewarming phase, d-ROM levels increased gradually in the temperature range from 34°C to 35°C, while from 35°C to 36°C they increased in a more pronounced manner from 294 U CARR to 403 U CARR.

As for the BAP level at 33°C, the BAP reading at 34°C remained low (1991 *μ*M). From 34°C to 35°C, BAP levels increased to 2223 *μ*M, while from 35 to 36°C they underwent an abrupt change from 2223 *μ*M to 3370 *μ*M. 

In response to TH, the patient's level of consciousness was completely restored. He was transferred from the ICU to a general ward for cardiovascular medicine 10 days after his admission.

## 4. Discussion

In the present study, we have demonstrated four important findings. (1) TH reduces d-ROM levels in patients with PCAS. (2) d-ROM levels increase during rewarming and are correlated with brain temperature. (3) TH suppresses BAP in patients with PCAS. (4) The BAP level also increases with rewarming and is correlated with brain temperature.

ROS appear to play an important role in various neuropathological conditions [26–28]. As it has been shown that oxidative stress induces lipid peroxidation and brain damage, one of the keys to manage the latter may be to control the former. Animal experiments have supported the notion that free radical scavengers and antioxidants dramatically reduce cerebral damage [24, 29–31]. Recently, the free radical scavenger edaravone (MCI-186) was shown to prevent lipid peroxidation under conditions that produce neurological damage [29, 30, 32, 33]. In this way, the control of oxidative stress is an important therapeutic strategy to combat various forms of acute neurological damage.

The direct measurement of ROS and free radicals in a standard laboratory is difficult owing to their biochemical instability. The authors have developed a direct method for monitoring free radicals in the blood using an ex vivo electron spin resonance (ESR) technique [16–18]. However, because ESR equipment is expensive and requires special facilities, the method has not yet been adopted for clinical examinations. FRAS, on the other hand, is a simple analytical system used to measure oxidative stress. The main component of ROMs is hydroperoxides, which, despite their oxidation capacity, are relatively stable in the blood compared to their parent free radicals, thus facilitating their detection.

The d-ROM test is recognized as being useful for the evaluation of oxidative stress in a clinical setting [22, 34–38]. In the present study, pre-TH d-ROM level was 333.6 ± 79.0 U CARR, which is slightly higher than that of reference values from healthy subjects [19], although reported values for healthy subjects vary significantly [35, 38, 39]. Komatsu et al., for example, reported that lifestyle and daily food intake affect d-ROM levels [39]. They reported that d-ROMs level in healthy Japanese people (*n* = 220, 21–98 y) was 335.3 ± 59.8, which is similar to the pre-TH d-ROM levels that we measured. It is unclear why the pre-TH d-ROM levels in our patients were not elevated, but it could be due to the fact that the pre-TH blood sample was obtained several hours after the induction of ROSC and that body temperature was maintained under 35°C (normothermia) through the pre-TH phase. It has been reported that the blood d-ROM levels gradually elevated after brain injury in mice [24]. And there were no significant changes between control and 6 hours after brain injury. This data indicated that pre-TH d-ROM levels did not dramatically change compared to healthy Japanese people. In this present study, the authors reported that, given that TH suppressed d-ROM level and that levels increased during the rewarming phase, d-ROM monitoring could thus be useful in a clinical setting for evaluating the effectiveness of TH as an antioxidative treatment for patients with PCAS. Several papers have previously reported that the d-ROM test could be useful for evaluating the effectiveness of antioxidative treatment in a clinical setting [38, 40, 41]. 

Recently, the use of mild “therapeutic” hypothermia (32–34°C) during the 24 to 36 hours after resuscitation was found to improve survival and neurological recovery in comatose survivors of cardiac arrest [11, 12]. The therapeutic target of TH was thought to be the reduction of I/R-induced injury to the brain. Recently, the therapeutic target has not only been to reduce I/R injury to the brain, but also to reduce injury to other organs [42–45]. The control of oxidative stress is now seen to be an important factor underlying the TH-induced reduction of brain damage involving I/R injury. Previous studies at both the laboratory and clinical levels have shown that TH reduces oxidative stress. For example, Baiping et al. reported that mild hypothermia decreased lipid peroxidation in the dog cerebral cortex 2 h after ROSC [13], while Kuo et al. demonstrated that brain cooling accomplished by the infusion of 4°C saline reduced oxidative stress after TBI in the rat [46]. In a clinical setting, Krüger et al. reported a reduction in nitrotyrosine and nitrate/nitrite levels during mild TH compared with the normothermic period in cardiac arrest survivors [47]. Mihara et al. on the other hand demonstrated, using ex vivo ESR, that mild TH suppressed alkoxyl radical levels in patients with TBI compared with the normothermic therapy [16]. In contrast to the above, some studies have referred to altered biodefence activity resulting from oxidative stress in TH. For example, Kuo et al. reported that the activities of glutathione peroxidase, glutathione reductase, superoxide dismutase (SOD), and catalase in hypothermic rats were higher than those in normothermic rats after TBI [46]. Gong et al. reported that mild hypothermia attenuates mitochondrial oxidative stress in the cerebral cortex, which may be associated with the enhancement of MnSOD activity and expression via Nrf2 activation after I/R injury [48]. In both these papers, the evaluation of biodefence activity was performed at different time points after rewarming. In the present study, however, we evaluated oxidative stress and antioxidant potential through all phases of the TH protocol. A proper evaluation of oxidative stress is obtained by measuring the relative balance of oxidative stress and biological antioxidant potential [21, 49], meaning that the measurement of BAP should always form part of this process. 

The rewarming phase is regarded as a highly sensitive phase in most TH procedures in a clinical setting, with critical side effects of TH such as the elevation of ICP and neuroinflammation commonly seen during this period [50]. We found that d-ROM and BAP levels increased along with rewarming and correlated with brain temperature. The current recommendation is to rewarm the patients after TH at a maximum rate of 0.25–0.5°C/h [51], though the optimal rewarming rate is unknown [4, 51, 52]. Bouwes et al. reported that a trend towards an association with poor outcome was seen in patients who were rewarmed at ≥0.5°C/h after cardiac arrest. A poor outcome was found in 15/21 patients (71%) with a high rewarming rate, compared to 54/103 patients (52%) with a normal rewarming rate (*P* = 0.08) [53]. In the present study, although the rewarming rate was low (0.1°C/h, <1°C/day), the BAP level in the rewarming period remained depressed, which suggests that antioxidant administration may be indicated during the rewarming phase to control neuroinflammation and oxidative stress.

A limitation of this study is that we were unable to clarify the mechanism underlying the decline of BAP in TH. One animal study showed that serum BAP correlated with serum *α*-tocopherol concentrations after TBI [24]. The activities of enzymatic antioxidants, such as SOD and catalase, are also decreased with hypothermia [54]. Alva et al. reported that F1,6BP was protective against the oxidative stress induced after rewarming by decreasing lipid peroxidation in plasma and by potentiating antioxidant enzyme activities in erythrocytes from the rat [55]. Additional clinical studies will be needed to clarify this point.

## 5. Conclusion

We have demonstrated that TH suppresses ROM production in a clinical setting. In contrast, however, TH also led to the suppression of BAP, even during the early phases of rewarming. Oxidative stress and antioxidant potential monitoring is useful for evaluating the efficacy of TH in PCAS patients and for the ongoing design and refinement of treatment strategies.

## Figures and Tables

**Table 1:** Patient characteristics.

Baseline characteristics	
All patients (*n* = 19)	
Sex, male (%)	16 (84.2)
Age, (median)	25–83 (53)
OHCA, *n* (%)	19 (100)
First rhythm	
VF	6 (31.6)
Non-VF/VT	13 (68.4)
Diagnosis	
AMI, *n* (%)	7 (36.8)
Others, *n* (%)	12 (63.2)
PCI, *n* (%)	7 (36.8)
Outcome at discharge, *n* (%)	
Death	4 (21.1)
Vegetative state	1 (5.3)
Severe disability	3 (15.8)
Moderate disability	1 (5.3)
Good recovery	10 (52.6)

OHCA: out-of-hospital cardiac arrest.

VF: ventricular fibrillation; AMI: acute myocardial infarction.

PCI: percutaneous coronary intervention.

## Abbreviations

BT: Brain temperature
ICP: Intracranial pressure
I/R: Ischemia/reperfusion
MnSOD: Manganese superoxide dismutase
ROS: Reactive oxygen species
SOD: Superoxide dismutase
TBI: Traumatic brain injury
TH: Therapeutic hypothermia
PCAS: Post cardiac arrest syndrome
ROSC: Return of spontaneous circulation.
